# Sampling From the Proteome to the Human Leukocyte Antigen-DR (HLA-DR) Ligandome Proceeds *Via* High Specificity* [Fn FN2]

**DOI:** 10.1074/mcp.M115.055780

**Published:** 2016-01-13

**Authors:** Geert P. M. Mommen, Fabio Marino, Hugo D. Meiring, Martien C. M. Poelen, Jacqueline A. M. van Gaans-van den Brink, Shabaz Mohammed, Albert J. R. Heck, Cécile A. C. M. van Els

**Affiliations:** From the ‡Institute for Translational Vaccinology, P.O. Box 450, 3720 AL Bilthoven, the Netherlands;; §Biomolecular Mass Spectrometry and Proteomics, Bijvoet Center for Biomolecular Research and Utrecht Institute for Pharmaceutical Sciences, Science Faculty, Utrecht University, Padualaan 8, 3584 CH Utrecht, the Netherlands;; ¶Netherlands Proteomics Centre, Padualaan 8, 3584 CH Utrecht, the Netherlands;; ‖Centre for Infectious Disease Control, National Institute for Public Health and the Environment, P.O. Box 1, 3720 AL Bilthoven, the Netherlands;; **Chemistry Research Laboratory, Department of Chemistry, University of Oxford, Mansfield Road, OX13TA, Oxford, United Kingdom;; ‡‡Department of Biochemistry, University of Oxford, South Parks Road, OX1 3QU, Oxford, United Kingdom

## Abstract

Comprehensive analysis of the complex nature of the Human Leukocyte Antigen (HLA) class II ligandome is of utmost importance to understand the basis for CD4^+^ T cell mediated immunity and tolerance. Here, we implemented important improvements in the analysis of the repertoire of HLA-DR-presented peptides, using hybrid mass spectrometry-based peptide fragmentation techniques on a ligandome sample isolated from matured human monocyte-derived dendritic cells (DC). The reported data set constitutes nearly 14 thousand unique high-confident peptides, *i.e.* the largest single inventory of human DC derived HLA-DR ligands to date. From a technical viewpoint the most prominent finding is that no single peptide fragmentation technique could elucidate the majority of HLA-DR ligands, because of the wide range of physical chemical properties displayed by the HLA-DR ligandome. Our in-depth profiling allowed us to reveal a strikingly poor correlation between the source proteins identified in the HLA class II ligandome and the DC cellular proteome. Important selective sieving from the sampled proteome to the ligandome was evidenced by specificity in the sequences of the core regions both at their N- and C- termini, hence not only reflecting binding motifs but also dominant protease activity associated to the endolysosomal compartments. Moreover, we demonstrate that the HLA-DR ligandome reflects a surface representation of cell-compartments specific for biological events linked to the maturation of monocytes into antigen presenting cells. Our results present new perspectives into the complex nature of the HLA class II system and will aid future immunological studies in characterizing the full breadth of potential CD4^+^ T cell epitopes relevant in health and disease.

Human Leukocyte Antigen (HLA)^1^ class II molecules on professional antigen presenting cells such as dendritic cells (DC) expose peptide fragments derived from exogenous and endogenous proteins to be screened by CD4^+^ T cells (1, 2). The activation and recruitment of CD4^+^ T cells recognizing disease-related peptide antigens is critical for the development of efficient antipathogen or antitumor immunity. Furthermore, the presentation of self-peptides and their interaction with CD4^+^ T cells is essential to maintain immunological tolerance and homeostasis (3). Knowledge of the nature of HLA class II-presented peptides on DC is of great importance to understand the rules of antigen processing and peptide binding motifs (4), whereas the identity of disease-related antigens may provide new knowledge on immunogenicity and leads for the development of vaccines and immunotherapy (5, 6).

Mass spectrometry (MS) has proven effective for the analysis HLA class II-presented peptides (4, 7, 8). MS-based ligandome studies have demonstrated that HLA class II molecules predominantly present peptides derived from exogenous proteins that entered the cells by endocytosis and endogenous proteins that are associated with the endo-lysosomal compartments (4). Yet proteins residing in the cytosol, nucleus or mitochondria can also be presented by HLA class II molecules, primarily through autophagy (9–11). Multiple studies have mapped the HLA class II ligandome of antigen presenting cells in the context of infectious pathogens (12), autoimmune diseases (13–17) or cancer (14, 18, 19), or those that are essential for self-tolerance in the human thymus (3, 20). Notwithstanding these efforts, and certainly not in line with the extensive knowledge on the HLA class I ligandome (21), the nature of the HLA class II-presented peptide repertoire and particular its relationship to the cellular source proteome remains poorly understood.

To advance our knowledge on the HLA-DR ligandome on activated DC without having to deal with limitations in cell yield from peripheral human blood (12, 21, 22) or tissue isolates (3), we explored the use of MUTZ-3 cells. This cell line has been used as a model of human monocyte-derived DCs. MUTZ-3 cells can be matured to act as antigen presenting cells and express then high levels of HLA class II molecules, and can be propagated *in vitro* to large cell densities (23–25). We also evaluated the performance of complementary and hybrid MS fragmentation techniques electron-transfer dissociation (ETD), electron-transfer/higher-energy collision dissociation (EThcD) (26), and higher-energy collision dissociation (HCD) to sequence and identify the HLA class II ligandome. Together this workflow allowed for the identification of an unprecedented large set of about 14 thousand unique peptide sequences presented by DC derived HLA-DR molecules, providing an in-depth view of the complexity of the HLA class II ligandome, revealing underlying features of antigen processing and surface-presentation to CD4^+^ T cells.

## EXPERIMENTAL PROCEDURES

### 

#### 

##### Ethics

Institutional principles of RIVM relating to the use of material and data obtained from human subjects, including prototype cell lines obtained via cell bank catalogues, are in agreement with the guidelines expressed for Good Clinical Practice expressed in the Declaration of Helsinki.

##### Cell Culturing, Lysis of Cells and Isolation of HLA Class II-associated Peptides

The deposited MUTZ-3 cell line, a human HLA-DR10, -DR11, -DR52 (HLA-DRB1*10, HLA-DRB1*11, HLA-DRB3*01) positive acute myelo-monocytic leukemia serving as a dendritic cell model (kindly provided by Dr. R. Scheeper, VU University Medical Center, Amsterdam), was grown under maintenance conditions in roller bottles in α-Minimum Essential Medium (Gibco, Thermo Fisher Scientific, Bremen, Germany), supplemented 20% heat-inactivated FBS (Hyclone, Logan, USA), 100 U/ml penicillin, 100 μg/ml streptomycin (Gibco), 2 mm l-glutamine (Gibco), and 25 U/ml GM-CSF (Pepotech, London, UK)^1^. MUTZ-3 cells were induced into an immature DC state by a 5-day exposure to 1000 U/ml GM-CSF (100 ng/ml), 1000 U/ml IL-4 (20 ng/ml) and 2.5 ng/ml TNF-α. Immature MUTZ-3 DC were matured by increasing the concentration of TNFα to 75 ng/ml for 20 h. During this maturation phase BCG antigens were present as an antigenic pulse (kindly provided by Camille Locht, Institut Pasteur de Lille, France). The mature state was verified based on the expression of DC-associated maturation markers CD40, CD80, CD83, and CD86 by flow cytometry (data not shown). The large bulk of 1.2 × 10^9^ cells stimulated MUTZ-3 was washed in ice cold PBS and snap frozen before lysing and solubilizing of cell membrane proteins with Nonidet P40 containing IP lysisbuffer (Thermo Fisher Scientific). After removal of the nonsolubilized fraction using ultracentrifugation, HLA class II molecules were immunoprecipitated from the MUTZ-3 cell lysate using the HLA-DR-specific monoclonal antibody L243. An aliquot of the MUTZ-3 cell lysate after HLA-DR pull down was used for proteomics. HLA class II molecules and associated peptides were eluted with 10% acetic acid and peptides were collected by passage over a 10-kDa mw cutoff membrane and concentrated using vacuum centrifugation.

##### MUTZ-3 Cell Lysate Digestion

The MUTZ-3 cell lysate was diluted in 2 m urea, 50 mm ammonium bicarbonate containing one tablet of EDTA-free protease inhibitor mixture (Sigma-Aldrich, Zwijndrecht, The Netherlands) and one tablet of PhosSTOP phosphatase inhibitor mixture (Roche, Almere, The Netherlands). Cysteine residues were reduced and alkylated using 200 mm dithiotreitol (Sigma-Aldrich) and 200 mm iodoacetamide (Sigma-Aldrich). The proteins were digested with Lys-C (Roche) at an enzyme/protein ratio of 1:75 for 4 h at 37 °C. Two times diluted samples were digested with trypsin (Roche) overnight at 37 °C at an enzyme/protein ratio of 1:100. Peptide mixtures were desalted using a 1-cc Sep Pack C18 columns (Waters, Etten-Leur, The Netherlands) according manufacture's protocol.

##### Fractionation of HLA-DR Ligands and Tryptic Peptides

HLA-DR eluted peptides were fractionated by strong cation exchange (SCX) chromatography (27). The system consists of a Hypercarb™ trapping column (200 μm I.D., 5 mm, 7 μm particle size, Thermo Fisher Scientific) and SCX analytical column (200 μm I.D., 12 cm, polysulfoethyl aspartamide, 5 μm, PolyLC, Columbia, USA). The peptides were separated by a linear salt gradient ramping to 500 mm KCl in 0.1 m HOAc and 35% acetonitrile at a column flow rate of 2 μl/min. A total number of 26 fractions (2 min per fraction) were collected, dried down using a vacuum centrifuge, and reconstituted. Based on the LC-MS/MS signal intensities of pre-analyzed sample aliquots the 10 most informative fractions were selected for analysis.

Tryptic peptides from the MUTZ-3 digest were fractioned by SCX using a ZorbaxBioSCX-Series II column (0.8 mm I.D., 50 mm, 3.5 μm particle size, Agilent Technologies, Waldbronn, Germany). A multistep gradient up to 500 mm Nacl in 0.05% formic acid 20% acetonitrile was used to separate the tryptic peptides (28). Fractions were pooled based on their UV signal intensity to a total of 10 fractions.

##### Reversed Phase Liquid Chromatography and Mass Spectrometry

For the HLA ligands, each individual SCX fraction was analyzed in triplicate by nanoscale LC-MS using a Thermo Scientific EASY-nLC 1000 (Thermo Fisher Scientific) and ETD enabled LTQ Orbitrap Elite mass spectrometer (Thermo Fisher Scientific) with either EThcD, HCD or ETD fragmentation. The system comprises an in-house packed 20 mm × 100 μm ID trapping column (Reprosil C18, 3 μm, Dr Maisch, Ammerbuch, Germany) and a 50 cm × 50 μm ID analytical column (Poroshell 120 EC C18, 2.7 μm, Agilent Technologies) heated to 40 °C. The gradient for the separation linearly ranged from 7% to 30% of solvent B in 90 min at a flow rate of 100 nl/min. The column effluent was directly electro-sprayed into the MS using a gold-coated fused silica tapered tip of ∼5 μm I.D. Full MS spectra (*m*/*z* 300 to 1,500) were acquired in the Orbitrap at 60,000 resolution (FWHM). The 10 most abundant precursor ions were selected for either data-dependent EThcD, HCD or ETD fragmentation (isolation width of 1.5 Th) at an abundance threshold of 500 counts. Fragment ions were detected in the Orbitrap analyzer at 15,000 resolution (FWHM). The automatic gain control (AGC) target in MS/MS was set to 3 × 10^5^ for EThcD, 7 × 10^4^ for HCD, and 1 × 10^5^ ETD. The maximum ion accumulation time for MS scans was set to 250 ms and for MS/MS scans to 1500 ms. For EThcD, modified instrument firmware was used to allow all-ion HCD fragmentation after an initial ETD. The HCD normalized collision energy was set to 32%. The ETD reaction time was set to 50 ms and supplemental activation and charge dependent activation time was enabled. Precursor ions with unknown and +1 charge states were excluded from MS/MS analysis. Dynamic exclusion was enabled (exclusion size list 500) with a repeat count of 1 and an exclusion duration of 60 s.

The SCX fractions of the tryptic digested MUTZ-3 cells were analyzed by LC-MS/MS using an Agilent 1290 Infinity System (Agilent Technologies) modified for nanoflow LC (passive split) connected to a TripleTOF analyzer (Sciex, Nieuwerkerk aan den Ijssel, The Netherlands). Peptides were eluted using a similar trapping and analytical column system and LC gradient conditions as described above. A voltage of 2.7 kV was applied to the needle. The survey scan was from 375 to 1250 *m*/*z* and the high resolution mode was utilized, reaching a resolution of up to 40,000. Tandem mass spectra were acquired in high sensitivity mode with a resolution of 20,000. The 20 most intense precursors were selected for subsequent fragmentation using an information dependent acquisition, with a minimum acquisition time of 50 ms.

##### Data Analysis

The raw files collected from the TripleTOF were first recalibrated based on five background ions with *m*/*z* values of 391.2847, 445.12003, 51913882, 593.15761, 667.17640. The calibrated raw files were converted to mgf by the AB Sciex MS Data Converter (version 1.3 beta) program before analysis with Proteome Discoverer 1.4. RAW files acquired with the Orbitrap Elite were directly analyzed with Proteome Discoverer 1.4 software package (Thermo Fisher Scientific) using default settings unless otherwise stated. For the EThcD and ETD spectra the nonfragment filter was added with the following settings: The precursor peak was removed within a 1 Da window, charged reduced precursors and neutral loss peaks were removed within a 0.5 Da window. MS/MS scans were searched against the human Uniprot database (2012, 20,205 entries) using the SEQUEST HT mode (Proteome Discoverer 1.4, Thermo Fisher Scientific). Precursor ion and MS/MS tolerances were set to 3 ppm and 0.02 Da, respectively. In SEQUEST, spectrum matching was set to one for c and z ions for ETD data, b and y ions for HCD and b, y, c, and z for EThcD. The data were searched with no enzyme specificity, methylation and dimethylation (Arg, Lys) acetylation (N terminus, Lys), cysteinylation (Cys), deamidation (Asn, Gln, Arg) and oxidation (Met) set as variable modifications. The allowed peptide length was set between 6 and 30 amino acids, the typical length distribution of HLA class II peptides. An additional search was performed with no enzyme specificity and phosphorylation (Ser, Thr, Tyr), deamidation (Asn), and oxidation (Met) set as dynamic modification. PTM assignments were validated manually. The data sets were searched (separately) against the full reversed database, and the Percolator software was used to rescore and filter the peptide-to-spectrum matches (PSM) to a < 1% false discovery rate (FDR) (29). SEQUEST searching combined with Percolator is particularly useful for the analysis of EThcD data and boosts the performance of HLA ligandome identification while maintaining stringency, as validated elsewhere (30). The peptide identification list was additionally filtered for Xcorr score ≥ 1.5. The final refiltered peptide identification list was used as the HLA-DR ligandome for further analysis.

TripleTOF data files were analyzed using identical settings unless otherwise stated. Precursor ion tolerance was set to 20 ppm and the MS/MS tolerance to 0.15 Da. In SEQUEST, spectrum matching was set to 1 for y and b ions. The data were searched with specificity for trypsin and enabling 2 miss cleavages. Oxidation (Met), N-terminal acetylation, phosphorylation (Ser, Thr, Tyr), methylation (Arg, Lys), dimethylation (Arg, Lys) were set as dynamic modification and carbamidomethylation (Cys) was set as a static modification.

The amount of HLA-DR peptides presented at the cell surface expressed as copy number per cell were estimated based on the MS intensities provided by proteome discoverer and known amounts of the synthetic peptides angiotensin-III and oxytocin, which were spiked in each fraction prior to LC-MS analysis. CELLO2GO (31) was used for protein subcellular localization prediction. The peptide binding affinities and the 9 a.a. binding core for HLA-DR10, HLA-DR11, and HLA-DR52 were predicted using the NetMHpan-3.0 algorithm (32). Peptides with a moderate to high binding affinity (IC) < 1000 nm were considered as potential binder for a particular allele. The GibbsCluster-1.0 algorithm (33) was for simultaneous alignment and clustering of complete date set of HLA-DR-associated peptides. Gibbs clustering was performed using default settings (33), with preference of hydrophobic amino acids at position P1, the number of clusters set to 1–4, λ set to 0.8. Sequence logo's we created using IceLogo (34), with *p* values set to 0.005. Following accepted procedures the protein/peptide abundances were estimated based on their spectral counts normalized to the protein length (35). The mass spectrometry data have been deposited to the ProteomeXchange Consortium (http://proteomecentral.proteomexchange.org) via the PRIDE partner repository (36) with the data set identifier PXD002951.

## RESULTS

### 

#### 

##### Identification of Naturally Presented HLA-DR-peptides

**Fig. 1.**
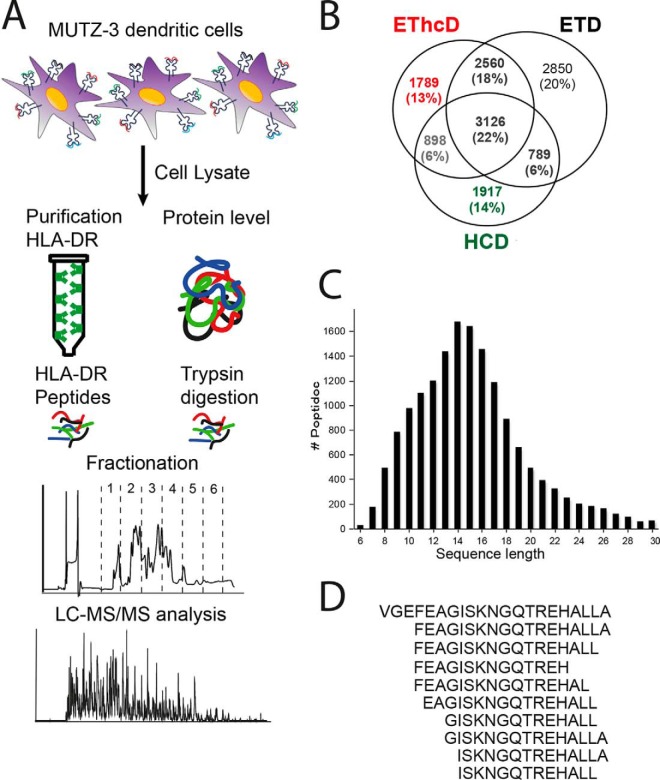
**HLA-DR ligandome analysis and characteristics.** *A*, Workflow for the analysis of the HLA-DR-presented ligandome and proteome of MUTZ-3 DC. For the ligandome, following affinity purification of HLA-DR-peptide complexes, peptides were released by acid elution, fractionated by strong cation exchange (SCX) and analyzed by LC-MS/MS using ETD, EThCD, and HCD fragmentation. Global proteome analysis was performed following trypsin digestion of lysed MUTZ-3 cells, using SCX fractionation prior to LC-MS/MS analysis. *B*, Venn diagram displaying the overlap and unique contribution to the identified HLA-DR ligandome by EThcD, ETD and HCD. *C*, Peptide length distribution in the combined HLA-DR ligandome (*n* = 13,918). *D*, Illustrative nested set of HLA-DR-associated peptides derived from Elongation factor 1-α 1, consisting of 10 peptide length variants with a consensus core binding motif but varying extended N- and C termini.

HLA-DR-peptides were isolated from human matured MUTZ-3 DCs and prefractionated by strong cation exchange chromatography prior to analysis by reversed phase LC-MS/MS, employing the complementary peptide fragmentation methods HCD, ETD, and EThcD (Fig. 1*A*). Table I summarizes the global outcome of the HLA-DR peptide identification results (supplemental Data). Although the highest number of MS/MS events were acquired by HCD, it showed a substantial lower identification rate than ETD and EThcD. In our work by using ETD the largest number of unique peptides could be identified (9431), followed by EThcD (8254), and finally HCD (6725). The combination of the three techniques was essential to expand the ligandome identification results, because only 53% of all peptides were identified by two or more fragmentation techniques (Fig. 1*B*). The combined results yielded 13,918 unique HLA-DR-peptides originating from just under two thousand (1980) source proteins (Table I).

**Table I:** Comprehensive HLA-DR ligandome analysis of mature MUTZ-3 DC by mass spectrometry using the complementary peptide fragmentation techniques ETD, HCD, and EThcD

	ETD	HCD	EThcD	Total
Data set				
Total # of MS/MS spectra	54,059	61,129	46,351	161,539
peptide-to-spectrum-matches, PSMs (<1% FDR)	14,100	10,694	12,755	37,549
identification rate (%)	26	17	28	23
unique HLA-DR peptides	9431	6725	8254	13,918
unique source proteins	1516	1314	1280	1980
Characteristics of the HLA-DR ligandome				
‘nested sets’	1472	1066	1237	2060
peptides presented in nested sets	7624	5382	6998	11,278
median (and range) of peptide copy numbers per cell	2 (<1–596)	1 (<1–382)	3 (<1–962)	2 (<1–962)
median (and range) of unique HLA-DR peptides per source protein	2 (1–612)	2 (1–445)	2 (1–598)	2 (1–800)
median (and range) of nested sets per source protein	1 (0–38)	1 (0–32)	1 (0–36)	1 (0–44)
median (and range) of cumulative peptide copy number per source protein per cell	4 (<1–4085)	2 (<1–1958)	6 (<1–6610)	3 (<1–7450)

To check whether peptide identification depended on the peptide's physicochemical properties, we next evaluated the performance of HCD, ETD, and EThcD in terms of peptide length and charge state. Supplemental Fig. S1 shows that there were moderate differences in the physicochemical properties of the peptides identified by the applied peptide fragmentation techniques. EThcD displayed a preference for smaller (6–11 amino acids in length) and doubly charged peptides, whereas ETD displayed a bias for longer peptides with higher charge state.

The inventory of 13,918 HLA-DR peptides included peptides that contained oxidized methionine, deamidated asparagine, and glutamine, most likely modifications that are associated with sample preparation (613 modified peptides in total). We identified an additional fraction of 916 unique peptides bearing a variety of post translational modifications, including cysteinylation, N-terminal and lysine acetylation, serine and threonine phosphorylation, and (di)methylation on arginine and lysine residues.

When compared with some of the most extensive previous HLA class II studies in literature, our data set represents a substantial improvement of the number of HLA-DR peptides identified (supplemental Table S1). Despite the limited overlap between peptides identified, likely because of different HLA-DR backgrounds, our study covered a large fraction (∼50%) of the previous identified source proteins and extensively increased the list of protein antigens presented by HLA class II molecules.

##### Global Characteristics of the HLA-DR Ligandome

The 13,918 HLA-DR peptides identified in the total data set ranged in length from six up to 30 amino acids (Fig 1*C*), with a preference for 14–17 residues (50%) (8). The vast majority of peptides (84%) corresponded to so-called nested sets, having a common binding core extended by flanking N- and/or C-terminal residues (Table I, Fig. 1*D*). In total, 2060 nested sets were observed, an average of ∼1 nested set per protein (Table I). The majority of nested sets were represented by two or three peptides but some consisted of more than 60 variants.

The NetMHCIIpan-3.0 algorithm (32) was used to predict the 9 amino acids core sequence of the peptides that facilitates binding to the expressed HLA-DR10, HLA-DR11 and HLA-DR52 molecules. A total of 10,393 binding cores were predicted to have a moderate to strong affinity to the expressed HLA-DR molecules (< 1000 nm). These predicted binding cores were derived from only 5795 unique peptides, indicating that for many peptide multiple core sequences were predicted to bind to different HLA-DR molecules and no single HLA-DR presenting molecule could be assigned. From the 10,393 predicted binding cores, only 1877 were predicted to bind to a single HLA-DR molecule, whereas the remaining ones displayed unambiguity and/or redundancy having a moderate to strong predicted affinity to multiple HLA-DR molecules. These results likely indicate that the NetMHCIIpan3.0 software lacks specificity to discriminate in binding between the three investigated different HLA-DR molecules. Therefore, we decided to also make use of the Gibbs alignment and clustering algorithm (Gibbs-Cluster-1.0) (33) to extract the binding motifs directly from the complete set of HLA-DR ligands. We clustered the complete set of peptides into four groups, one for each investigated HLA-DR molecule and one nonspecific cluster. The Gibbs-Cluster-1.0 could assign to the different HLA-DR clusters a total of 8569 unique peptides, without overlap between the generated peptide groups.

**Fig. 2.**
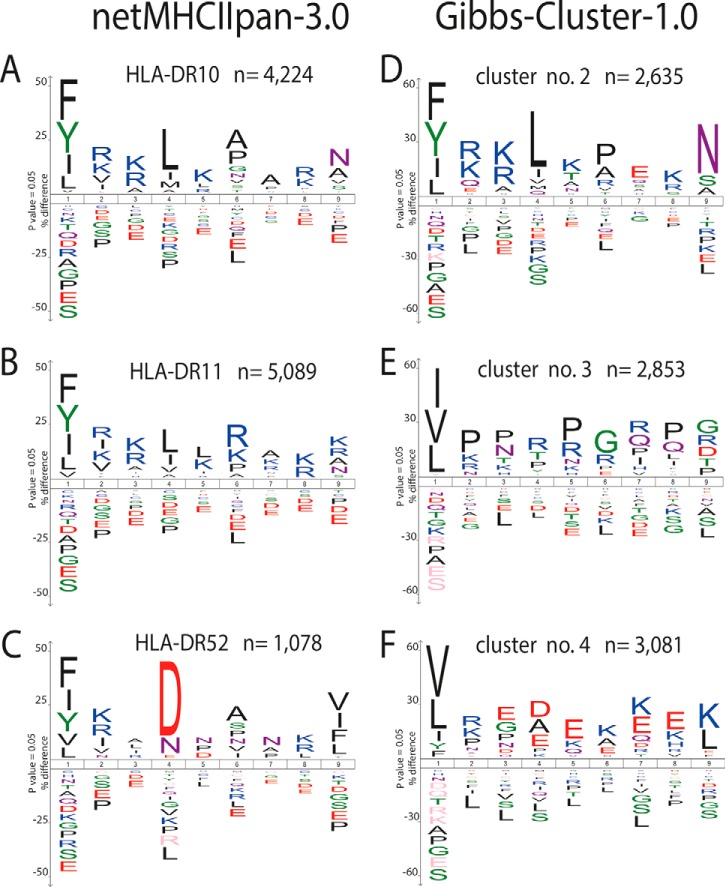
**Binding motifs extracted from the HLA-DR ligandome by either NetMHCIIpan3.0.** and by unsupervised clustering using Gibbs-Cluster-1.0. Sequence logo's are shown for the nine amino acid binding motif enabling binding to the expressed HLA-DR molecules. Anchor residues located on position P1, P4, P6, and P9 are known to be important for binding. *A–C*, consensus binding motifs from the complete set of HLA-DR ligands for HLA-DR10 (*A*), HLA-DR11 (*B*) and HLA-DR52 (*C*) as extracted by using NetMHCIIpan3.0. *D–F*, Unsupervised analysis by alignment and clustering analysis (using Gibbs-Cluster-1.0) reveals unique HLA-DR binding motifs. The inserts display the number of predicted 9 amino acid binding cores found that contribute to the shown sequence logo's.

Fig. 2 shows three sequence logo's generated by the Gibbs-Cluster-1.0 analysis (Fig. 2*D*, 2*E*, 2*F*), two of which (Fig. 2*D*, 2*F*) agree reasonably well with the sequence logo's of the NetMHCIIpan3.0 predictions for HLA-DR10 and HLA-DR52 (respectively Fig. 2*A*, 2*C*). The sequence logo's of the predicted binding motifs exhibited marked differences between HLA-DR10, HLA-DR11, and HLA-DR52 (Fig. 2) at the P1, P4, P6, and P9 anchor residues. All three expressed HLA-DR molecules had a clear selectivity for both aliphatic and aromatic hydrophobic residues such as Phe, Tyr, Leu, and Ile at one or more of the anchor positions. Although aspartic acid (Asp) was found exclusively enriched at anchor position P4 for HLA-DR52, the Gibbs cluster analysis indicated that the negatively charged glutamic acid (Glu) was preferred on P3, P4, and P5 as well. For all three HLA-DR molecules we found that the positively charged residues Lys and Arg were moderately enriched at the positions P2, P3, P5, and P8. This observation was confirmed by the Gibbs cluster analysis.

##### Cleavage Specificity of Proteases Involved in HLA-DR Ligand Processing

**Fig. 3.**
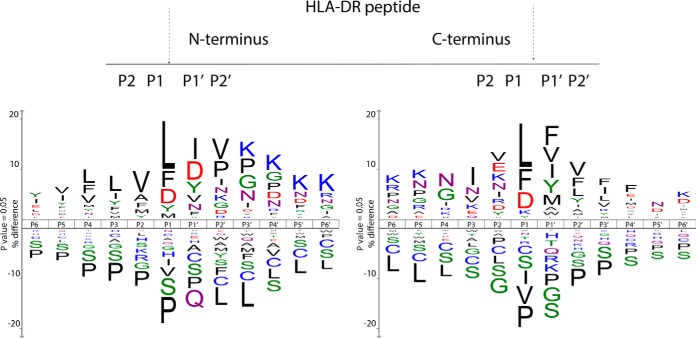
**Observed sequence specificity in the proteolytic processing of HLA-DR peptides.** Both the N-terminal and C-terminal cleavage specificity observed in HLA-DR ligand processing are shown. The sequence logo's depict six amino acids represented as P6…P1 and six amino acids as P1′…P6′, which are located at the N- (left) and C-terminal (right) scissile sites of HLA-DR-associated peptides, respectively. The residues that are statistically over-represented are shown on the upper part of the IceLogo, whereas under-represented at the lower part (95% confidence level).

To determine which endo-lysosomal proteases may be involved in the generation of the HLA-DR ligands, we analyzed the distribution of amino acids at the cleavage sites at both the N terminus and the C terminus of the ligands. Fig. 3 shows the over- and under-represented amino acids flanking the cleavage site both at the N terminus (left panel) and the C terminus (right panel). For both termini, cleavage preferentially occurs between hydrophobic and acidic residues. The high preference for Leu, Phe and Asp at P1 of the cleavage site hints to a dominant role of the proteases Cathepsin D or Cathepsin E (37, 38). In our subsequent global proteome analysis of MUTZ-3 cells (Fig 1*A*) a prominent expression of Cathepsin D compared with other proteases involved in the endo-lysosomal degradation, could be established (supplemental Table S2). The proteomic analysis also revealed the presence of several other proteases possibly involved in the ligand processing, such as Cathepsin B, H, S, Z, albeit generally detected at lower abundance than Cathepsin D. Therefore, Cathepsin D is likely the most prominent protease involved in the processing of MHC II molecules in MUTZ-3 cells.

**Fig. 4.**
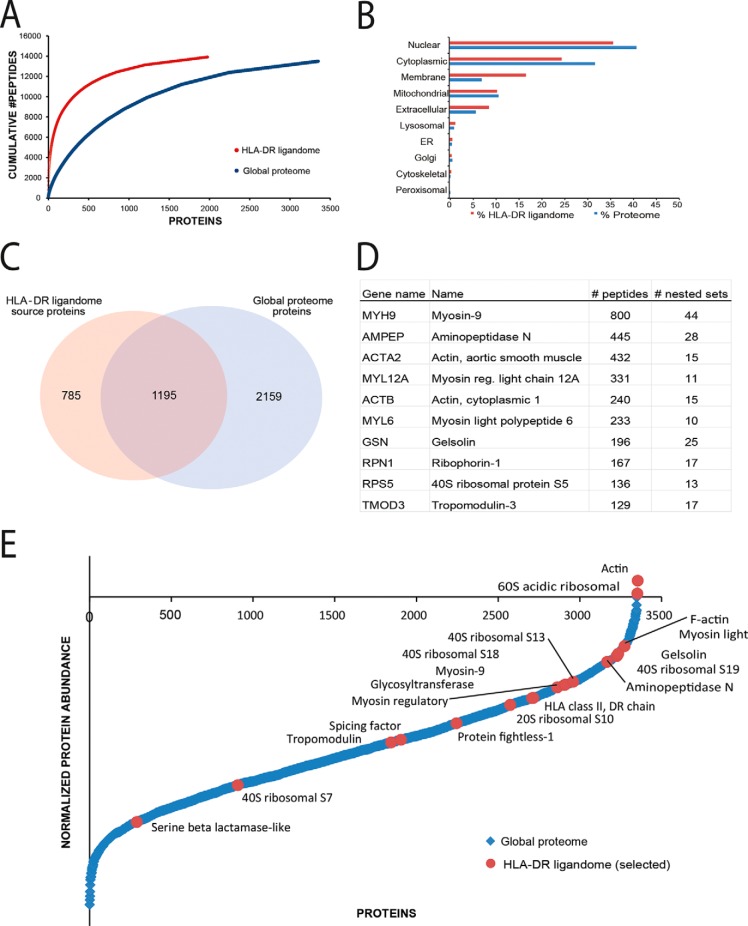
**Source proteome of the HLA-DR ligandome.** *A*, The cumulative number of unique peptides as function of the number of proteins identified by global proteome (blue) and the HLA-DR ligandome (red) analysis. Proteins are ranked from highest to the lowest number of representative peptides. *B*, The CELLO2GO algorithm was used to predict the subcellular localization of source proteins obtained by HLA-DR ligandome (blue bars) and global cellular proteome (red bars) analysis. *C*, Venn diagram showing the overlap between source-proteins identified in the HLA-DR ligandome and the full proteome. *D*, Source proteins most frequently sampled by HLA-DR with the number of observed unique HLA-DR peptides and number of nested sets. *E*, Dynamic range plot of the global cellular proteome of MUTZ-3 (blue dots). The assigned proteins (red dots) were the 19 out of 25 most abundantly expressed proteins in the HLA-DR ligandome (6 proteins HLA-DR-sampled proteins were not identified by global proteome analysis).

The specificity profiles also showed amino acid residues that were under-represented at the cleavage site (Fig. 3, lower parts of logo's). The small amino acids proline and serine were identified as poor substrates for the enzymes involved in antigen processing. Although proline residues were generally under-represented surrounding the cleavage sites, we found enrichment of proline residues close to the N-terminal end of HLA-DR ligands. Enrichment of proline residues located penultimate to the N-terminal cleavage has been linked to the blocking of N-terminal trimming in HLA class II peptides (39). This observation, linked to the identification of Aminopeptidase-N in both the global proteome and the HLA-DR ligandome, hints at a possible role for this enzyme in HLA class II processing in MUTZ-3 (supplemental Table S2, Fig. 4*D*).

##### High dominance in the HLA-DR binding groove

The median number of HLA-DR-peptides identified per source protein was 2, but varied widely ranging from a single peptide up to 800 unique peptides (Table I). The latter high number was found to be largely because of very extensive nested set formation. To obtain a (semi) quantitative impression of the HLA-DR sampling process, we calculated the peptide copy number per cell using known amounts of two synthetic peptides as reference which were spiked in each sample fraction prior to LC-MS analysis. These calculated copy numbers may however not accurately reflect the actual presentation levels because we do not account for losses during sample preparation and variation in MS detection (*e.g.* ionization efficiency). We estimated that HLA-DR peptides were presented on MUTZ-3 cells with an median of 2 copies/cell with a range between <1 and 962 copies/cell, indicating a dynamic range of four orders of magnitude in the display of potential individual CD4^+^ T cell epitopes (Table I). The surface presentation levels of the source proteins were determined by summing the copy per cell numbers of their representative peptides. The cumulative peptide copy number per protein per cell ranged from <1–7450 (5 orders of magnitude) (Table I), which follows directly from the high diversity in number of peptides presented per protein (*i.e.* nested sets). Based on these copy numbers, we estimated that the 100 most frequently sampled proteins already occupy ∼80% of the HLA-DR molecules, whereas the remaining 1881 proteins account for only ∼20% of the quantitative HLA-DR ligandome.

##### Selective Sampling of the Cellular Proteome by HLA-DR

The most frequently HLA-DR-sampled proteins included actins, myosins, aminopeptidase N and the HLA class II β chain itself (Fig. 4*D*, 4*E*). These proteins are known to be involved in cytoskeletal organization and antigen processing and presentation during maturation of monocytes into dendritic cells after stimulation (40, 41). We used PANTHER (42) for Gene Ontology (GO) classification of the HLA-DR ligandome, whereby the concomitantly analyzed MUTZ-3 cellular proteome was used as a reference set (Fig. 4*B*). Analysis of the GO terms of the source proteins of the HLA-DR ligandome against the background of the global MUTZ-3 proteome (rather than against the full human proteome) revealed an MHC class II pathway-specific signature in GO annotation for stimulated DCs. Compared with the cellular proteome, the HLA-DR ligandome was enriched in proteins involved in cell adhesion, immune system processes and system development (Supplemental Table 3). CELLO2GO (31) was used to predict protein cellular localization of the source proteins detected in the HLA-DR ligandome and the global cellular proteome (Fig. 4*B*). A major fraction of the ligandome source proteins originated from the nuclear compartment (36%), followed by the cytoplasm (25%) and the plasma membrane (17%). Compared with the MUTZ-3 global proteome, proteins contributing to the ligandome were relatively enriched in membrane associated proteins and proteins from the extracellular matrix, whereas proteins localized in the nucleus and cytoplasm were underrepresented.

Finally, we investigated the relation between the HLA-DR ligandome and the global MUTZ-3 proteome with respect to the identified proteins and their associated number of peptides. Global proteome analysis provided abundance estimates for about 3300 proteins (Fig. 4*A*). Only 1195 proteins (35%) were also identified as source proteins in the HLA-DR ligandome (Fig. 4*C*). Moreover, we observed that certain source proteins that were represented by a high number of HLA-DR peptides were absent in the global cellular analysis of MUTZ-3 cells, and *vice versa*, which could point to a rather weak correlation between expression levels of the HLA-DR ligandome and global proteome. To illustrate this, we determined the correlation between the proteins most frequently sampled by HLA-DR and their cellular expression levels (global proteome analysis) using a recently described peptide count approach (41). Fig. 4*E* shows that the most abundantly observed proteins in the HLA-DR ligandome are distributed over the full dynamic range plot of the source proteome.

## DISCUSSION

Natural processing and Human Leukocyte Antigen (HLA) class II presentation of extracellular and intracellular proteins are key yet still unpredictable functions of activated dendritic cells (DC) that steer and regulate adaptive immune responses. Advances in understanding the outcome of these processes require comprehensive analysis of endogenously processed and surface presented peptide repertoires, demanding cutting edge technology (43). To expand the mass spectrometry-based identification of HLA class II presented peptides we combined the (hybrid) peptide fragmentation techniques, HCD, ETD, and EThcD, on a large scale preparation of HLA-DR peptides derived from an *in vitro* matured human prototype monocyte-derived DC cell line, MUTZ-3. Cumulatively, the exceptionally high number of 13,918 unique identified HLA-DR peptides, a significant improvement in the number of ligands compared with previous endeavors (18, 22, 44, 45), gave unprecedented insight into various underlying features of selectivity in HLA-DR sampling of the ligandome.

MS-based identification of HLA class II-associated peptides by nonstandard ETD or EThcD based sequencing was until now little explored. Our data revealed an improved rate of peptide identification for techniques involving electron transfer dissociation over classical collision induced dissociation for HLA-DR peptides, confirming previous reports on the analysis of endogenous peptides (46, 47). HLA-DR-presented peptides were highly variable in nature, especially in their wide distribution in peptide length and charge state. ETD had a slight preferential bias for longer and/or higher charged HLA-DR-peptides (48, 49). Earlier, using identical nonstandard MS technology in an HLA class I peptide inventory (50) we identified 12,199 unique HLA class I peptides originating from 5,603 proteins in a human B lymphoblastoid cell line (*i.e.* on average ∼2 peptides per protein). In our present comprehensive survey addressing the HLA class II pathway we identified 13,918 HLA-DR ligands derived from only 1980 proteins (*i.e.* on average ∼7 peptides per protein). This much more sparse source proteome, together with the observation of many nested sets of length variants of the same epitope region, suggests that the overall HLA-DR epitope landscape available for surveillance by circulating CD4^+^ T cell populations is less diverse than the HLA class I ligandome inspected by CD8^+^ T cells.

The source proteins sampled by HLA-DR molecules reflected only a minor part (∼35% overlap) of the global proteome identified in the MUTZ-3 cell lysate. *Vice versa*, some most frequently sampled proteins in the HLA-DR ligandome were absent from the detectable MUTZ-3 proteome, which rather suggests a poor correlation between expression levels in the global proteome and protein representation in the HLA-DR ligandome. For the intrinsically different HLA class I antigen presentation route, multiple reports have established that it is difficult to predict ligand copy numbers from overall protein or RNA levels (21, 51–53), perhaps with the exception of proteins with high turn-over (35). Therefore, most studies including ours substantiate the selective sampling of proteome cargo for both HLA class I and class II molecules on their way to the cell surface. Indeed, GO analysis revealed that the surface-presented HLA-DR ligandome of MUTZ-3 DCs was biased for proteins involved in cell mobility, cytoskeletal re-organization, HLA class II processing and presentation, functions strongly linked to the maturation of monocytes into antigen presenting cells (40, 41). The MUTZ-3 DCs in our study were matured via TNFα, an endogenous “danger” signal that can induce the transition from an antigen capturing DC to an antigen presenting DC (54). Relative enrichment was also found for proteins associated with the membrane and extracellular matrix, the main contributors to the lysosomal degradation and HLA class II presentation pathway (55). Direct or indirect evidence for the dominance of membrane and extracellular proteins as source proteins for MHC class II ligandomes has been implied before (20, 56–58), in line with the mechanisms of endocytosis, lysosomal trafficking and processing, preceding MHC class II loading with peptides. Even while being underrepresented relative to the global MUTZ-3 proteome, proteins associated with the nuclear compartment or cytoplasm made up to 50% of the source proteins of the HLA-DR ligandome. We interpret this to be the outcome of autophagy, a stress response process shuttling large fractions of intracellular proteins into the lysosomal compartment promoting their presentation by HLA class II (59). Earlier autophagy has been implied to be essential for HLA-DR loading with intracellular material relevant for CD4^+^ T cells to monitor cellular virus infection (60, 61), transformation (62–64) or stress (65), or to shape their self-tolerance (10, 66, 67).

The observation that the most abundantly sampled source proteins occupied with their peptide sequences already ∼80% of the HLA-DR molecules, further indicates that in the antigen selection process certain proteins have strong advantage of being presented over others. The dynamic range of HLA-DR ligandome ranged from less than a single peptide copy per protein to extremes where cumulatively almost 7500 peptide copies per protein were detected per cell. Strong peptide hierarchies have been seen in the HLA class I ligandome (68) and human and murine MHC class II ligandomes of DC (69), and are essential for the recruitment and outcome of T cell responses (70, 71).

Our study substantiated the biochemical evidence that, apart from the cell biology of a particular type of antigen presenting cell in bringing together proteome and enzyme content in the MHC class II pathway, physical-chemical properties of protein sequences themselves play a dominant role in the high selectivity of proteome representation in HLA-DR. First, peptide sequences have to be generated but not destroyed by the proteolytic activity in a complex network of endo-lysosomal compartments of a particular cell (71, 72). Analysis of proteolytic sites located at the peptide N- and C terminus revealed that processing of HLA-DR ligands mainly involved cleavage between hydrophobic and/or acidic residues, corresponding to the substrate specificity of Cathepsin D (37, 38). Cathepsin D is known to be highly expressed is lysosomes, but how exactly this enzyme contributes to HLA class II peptide processing and presentation remains ambivalent as it can both generate and destroy T cell epitopes. Whether the here-observed dominant role of Cathepsin D in antigen processing is specifically associated with the TNFα maturation of the DCs needs further research. In another study Cathepsin S was reported to be more involved in the thymic HLA-DR peptidome (20).

Second, the created ligands need to meet any of the binding motifs of the HLA-DR allomorphs expressed by the cell in order to be presented. We complemented NetMHCII-pan3.0 predictions, that may struggle with allocating 9-mer binding cores in large sets of length variants, with Gibbs alignment and clustering analysis (Gibbs-Cluster-1.0) (33). With this latter algorithm, binding motifs were unbiasedly extracted from the complete set of HLA-DR-peptides, indicating the strong selectivity of the expressed HLA-DR molecules to bind peptides mainly through hydrophobic and aliphatic anchor residues. We also found an enrichment of positively charged residues (Lys, Arg) on nonanchor positions (P2, P3, P5, and P8). Selectivity for arginine on the P8 positions of HLA-DR10-associated peptides has been reported previously (45). Positively charged amino acids flanking the anchor residues could play an important role in the interaction of the HLA-DR-peptide complex with the T-cell receptor. Novellino *et al.* (73) showed that a melanoma-specific T-cell clone that interacts with the HLA-DR10-restricted peptide YFAAELPPRN lost recognition when arginine was substituted by glycine. Likely, ETD and EThcD have contributed to the unambiguous assignment of these positively charged amino acids as these fragmentation techniques are specifically beneficial for peptides harboring internal basic residues (49, 74).

In conclusion, complementary peptide fragmentation techniques allow the in depth profiling of human DC derived HLA class II-associated peptides. Our results, based on a single MUTZ-3 HLA-DR ligandome, pave the way for further exploration of HLA class II peptide repertoires under different maturation and antigenic conditions. Exposure of peptides at a wide dynamic range of abundances yet with a relatively limited diversity through strong selective mechanisms seem hallmarks of this pathway. Whether these here-observed characteristics of antigen processing and presentation are widely applicable to the HLA class II pathway in human DCs needs further investigation of various DC models and primary cell cultures. The challenge in future studies will be to quantitatively and qualitatively characterize the full breadth of potential CD4^+^ T cell epitopes relevant in health and disease, especially if hidden as minor specificities among the larger self ligandome.

## Footnotes

^1^ The abbreviations used are:

HLA: human leukocyte antigen
DC: dendritic cell
ETD: electron-transfer dissociation
EThcD: electron-transfer/higher-energy collision dissociation
GO: Gene Ontology
HCD: higher-energy collision dissociation
SCX: strong cation exchange.
